# Supplementary material for: Iterative improvement in the automatic modular design of robot swarms
Source: PeerJ Comput Sci. 2020 Dec 7;6:e322. doi: 10.7717/peerj-cs.322 (PMC7924708; doi:10.7717/peerj-cs.322)
Supplement: Supplemental Information 3 [file peerj-cs-06-322-s003.zip › argos3/doc/api/standalone/a00586_source.html]

ARGoS: plugins/simulator/entities/quadrotor\_entity.cpp Source File


- Main Page
- Related Pages
- Namespaces
- Classes
- Files

- File List
- File Members

# plugins/simulator/entities/quadrotor\_entity.cpp

Go to the documentation of this file.

```
00001 
00007 #include "quadrotor_entity.h"
00008 #include <argos3/core/simulator/space/space.h>
00009 
00010 namespace argos {
00011 
00012    /****************************************/
00013    /****************************************/
00014 
00015    CQuadRotorEntity::SPositionControlData::SPositionControlData(
00016       const CVector3& c_position,
00017       const CRadians& c_yaw) :
00018       Position(c_position),
00019       Yaw(c_yaw) {}
00020 
00021    /****************************************/
00022    /****************************************/
00023 
00024    CQuadRotorEntity::SSpeedControlData::SSpeedControlData(
00025       const CVector3& c_velocity,
00026       const CRadians& c_rot_speed) :
00027       Velocity(c_velocity),
00028       RotSpeed(c_rot_speed) {}
00029 
00030    /****************************************/
00031    /****************************************/
00032 
00033    CQuadRotorEntity::CQuadRotorEntity(CComposableEntity* pc_parent) :
00034       CEntity(pc_parent),
00035       m_eControlMethod(NO_CONTROL) {
00036       Reset();
00037    }
00038 
00039    /****************************************/
00040    /****************************************/
00041 
00042    CQuadRotorEntity::CQuadRotorEntity(CComposableEntity* pc_parent,
00043                                       const std::string& str_id) :
00044       CEntity(pc_parent, str_id),
00045       m_eControlMethod(NO_CONTROL) {
00046       Reset();
00047    }
00048 
00049    /****************************************/
00050    /****************************************/
00051 
00052    void CQuadRotorEntity::Reset() {
00053       m_sPositionControlData.Position.Set(0.0f, 0.0f, 0.0f);
00054       m_sPositionControlData.Yaw.SetValue(0.0f);
00055       m_sSpeedControlData.Velocity.Set(0.0f, 0.0f, 0.0f);
00056       m_sSpeedControlData.RotSpeed.SetValue(0.0);
00057    }
00058 
00059    /****************************************/
00060    /****************************************/
00061 
00062    REGISTER_STANDARD_SPACE_OPERATIONS_ON_ENTITY(CQuadRotorEntity);
00063 
00064    /****************************************/
00065    /****************************************/
00066 
00067 }
```

---

Generated on 10 Jul 2018 for ARGoS by 
 1.6.1
